# Comparison of Knee Articular Cartilage Defect Size Between Measurements Obtained on Preoperative MRI Versus During Arthrotomy

**DOI:** 10.1177/23259671231193380

**Published:** 2023-09-06

**Authors:** Jade Perry, Jan Herman Kuiper, Helen S. McCarthy, Paul Jermin, Peter D. Gallacher, Bernhard Tins, Sally Roberts

**Affiliations:** † The Robert Jones & Agnes Hunt Orthopaedic Hospital NHS Foundation Trust, Oswestry, Shropshire, UK.; ‡ The School of Pharmacy & Bioengineering, Keele University, Staffordshire, UK.; *Investigation performed at The Robert Jones and Agnes Hunt Orthopaedic Hospital NHS Foundation Trust, Oswestry, Shropshire, UK*

**Keywords:** articular cartilage, autologous cell therapy, defect size, knee, magnetic resonance imaging, osteoarthritis

## Abstract

**Background:**

Treatment decisions for cartilage defects are often based on lesion size. Magnetic resonance imaging (MRI) is widely used to diagnose cartilage defects noninvasively; however, their size estimated from MRI may differ from defect sizes measured during arthrotomy, especially after debridement to healthy cartilage if undergoing autologous chondrocyte implantation.

**Purpose/Hypothesis:**

The purpose of this study was to evaluate the reliability of 2 methods to assess knee cartilage defect size on preoperative MRI and determine their accuracy in predicting postdebridement defect sizes recorded during arthrotomy. It was hypothesized that defect size would be predicted more accurately by the total area of abnormal articular cartilage rather than the area of full-thickness cartilage loss as identified on MRI.

**Study Design:**

Cohort study (diagnosis); Level of evidence, 3.

**Methods:**

This study included 64 patients (mean age, 41.8 ± 9.6 years) who underwent autologous cell therapy. Each patient received a 3-T MRI at 6.1 ± 3.0 weeks before cell implantation. Three raters, a radiologist, a surgeon, and a scientist, measured (1) the full-thickness cartilage defect area and (2) the total predicted abnormal cartilage area, identified by an abnormal signal on MRI. Interrater reliability was assessed using the intraclass correlation coefficient (ICC). Actual pre- and postdebridement defect sizes were obtained from intraoperative surgical notes. Postdebridement surgical measurements were considered the clinical reference standard and were compared with the radiologist’s MRI measurements.

**Results:**

Eighty-seven defects were assessed, located on the lateral (n = 8) and medial (n = 26) femoral condyle, trochlea (n = 17), and patella (n = 36). The interrater reliability of the cartilage defect measurements on MRI was good to excellent for the full-thickness cartilage defect area (ICC = 0.74) and the total predicted abnormal cartilage area (ICC = 0.78). The median full-thickness cartilage defect area on MRI underestimated the median postdebridement defect area by 78.3%, whereas the total predicted abnormal cartilage area measurement underestimated the postdebridement defect area by 14.3%.

**Conclusion:**

Measuring the full-thickness cartilage defect area on MRI underestimated the area to treat, whereas measuring the total abnormal area provided a better estimate of the actual defect size for treatment.

Articular cartilage defects within the knee joint present a clinical and socioeconomic burden.^24^ The capacity for natural regeneration of articular cartilage is limited, due to its avascularity and lack of innervation, such that chondral/osteochondral defects often lead to premature osteoarthritis (OA).^9,29^ Full-thickness articular cartilage defects in the knee joint commonly arise in patients after chronic joint stress, acute trauma, or sports-related injuries.^33^ These defects often cause joint dysfunction through progressive pain, limiting mobility and sports participation,^33^ although it should be noted that full-thickness articular cartilage defects have also been found in asymptomatic subjects.^18^ In an attempt to assuage pathology-related symptoms, restore the anatomy and function of the articular surface, prevent additional cartilage damage, and begin the repair process, cartilage defects can undergo surgical treatment.^23,25^

These surgical treatments range from bone marrow stimulation techniques such as microfracture,^41^ to chondrogenic tissue replacement via autologous or allogeneic osteochondral grafts,^7^‘^21^ and to cell therapy approaches such as autologous chondrocyte implantation (ACI)^10^ and even nonbiological implants such as minimetal arthroplasties.^40^ Deciding upon the most appropriate surgical treatment for articular cartilage defects is multifaceted. Clinicians must take into consideration both patient- and lesion-specific parameters, including prior treatments, patient activity levels, defect size, and location.^14^ In the UK, the National Institute for Health and Care Excellence (NICE) only recommends ACI in the knee for patients who have not previously had surgical treatment to repair their articular cartilage defects, have minimal generalized joint OA damage and have a defect size >2 cm^2^.^34^ Therefore, accurately estimating the size of a defect is crucial for treatment planning.^11,12,14^ Accurate measurement of defect size is also useful as a prognostic factor when considering OA progression and in making predictions with regard to treatment success^31^ and reimbursement costs.^32,38^

Magnetic resonance imaging (MRI) is the optimal clinical imaging modality for noninvasive evaluation of cartilage lesions. It is commonly used to determine the size and depth of cartilage defects in order to choose the most appropriate treatment option.^3,21,41^ However, estimated and actual treated defect sizes often differ. Despite the ease with which MRI scans can be used to measure the zone of full-thickness cartilage loss, this may not accurately predict the actual size of lesion treated during surgery. For example, ACI requires debridement around the lesion to healthy cartilage for successful attachment of the patch, which can significantly increase the lesion size to be treated.^14^ Discrepancies are likely; therefore; if only full-thickness cartilage defects are measured at the outset. Thus, the approach employed to quantify the total abnormal area by the radiologist or surgeon is key to understanding defect dimensions to be treated before surgery.^25^

Previous work has questioned the accuracy of MRI sizing of articular cartilage defects.^12,20^ Gomoll et al^20^ used a cutoff for defining a lesion >50% thickness loss of cartilage. In the study presented here, we compared the full-thickness cartilage defect area and the total predicted abnormal cartilage area on MRI scans with the final defect size obtained at the time of surgery (arthrotomy) after surgical debridement. In addition, we aimed to determine the interrater reliability of the 2 measurements. We hypothesized that preoperative MRI measurements of the total predicted abnormal cartilage area (ie, including altered morphology and/or signal of the cartilage) would have a smaller bias when estimating the final defect size measured during arthrotomy compared with measuring only the full-thickness component.

## Methods

### Patients and ACI Procedure

This retrospective study used data from a randomized controlled trial of cell therapy for cartilage repair (11/WM/0175; ISRCTN98997175). Informed, written consent was obtained from each trial participant before enrollment, and the study was performed according to the guidelines of the Declaration of Helsinki (1964). Cartilage defect measurements were obtained from MRI scans and arthrotomy findings in the knee joints of 64 participants (mean age, 41.8 ± 9.6 years). Each patient received autologous cell therapy in the knee to repair chondral or osteochondral lesions using a 2-stage procedure as described by Richardson et al,^37^ performed by either of 2 surgeons (J.B.R. [n = 38] or P.G. [n = 26]).

During arthroscopic surgery (stage 1), articular cartilage that appeared macroscopically normal was harvested from a lesser weightbearing region of the joint and/or bone marrow aspirated from the iliac crest. In our onsite Good Manufacturing Practice–approved laboratory, chondrocytes were isolated from the cartilage harvest and bone marrow–derived mesenchymal stromal cells from the bone marrow aspirate. Both cell types were individually culture expanded in monolayer for approximately 21 days. The patients then underwent an arthrotomy (stage 2), where the defect was measured using a ruler or caliper both pre- and postdebridement, before culture-expanded autologous cells being implanted beneath a porcine collagen patch (Chondro-Gide; Geistlich Pharma). All defect sizes and defect locations obtained during arthrotomy were recorded on a knee diagram.^42^

### Magnetic Resonance Imaging

All study patients received a preoperative MRI, performed on a 3-T scanner unit (Skyra) using a dedicated 16-channel knee coil with a T1-weighted sagittal spin-echo sequence, a sagittal proton density–weighted turbo spin-echo fat-suppressed (PD-FS) sequence, a coronal and axial PD-FS sequence, and a 3-dimensional PD-FS sequence in the sagittal plane. T2-weighted star maps were calculated based on a sagittal gradient-echo T2-weighted sagittal sequence. MRI scans were performed at a mean of 6.1 ± 3.0 weeks before stage 2.

**Figure 1.**
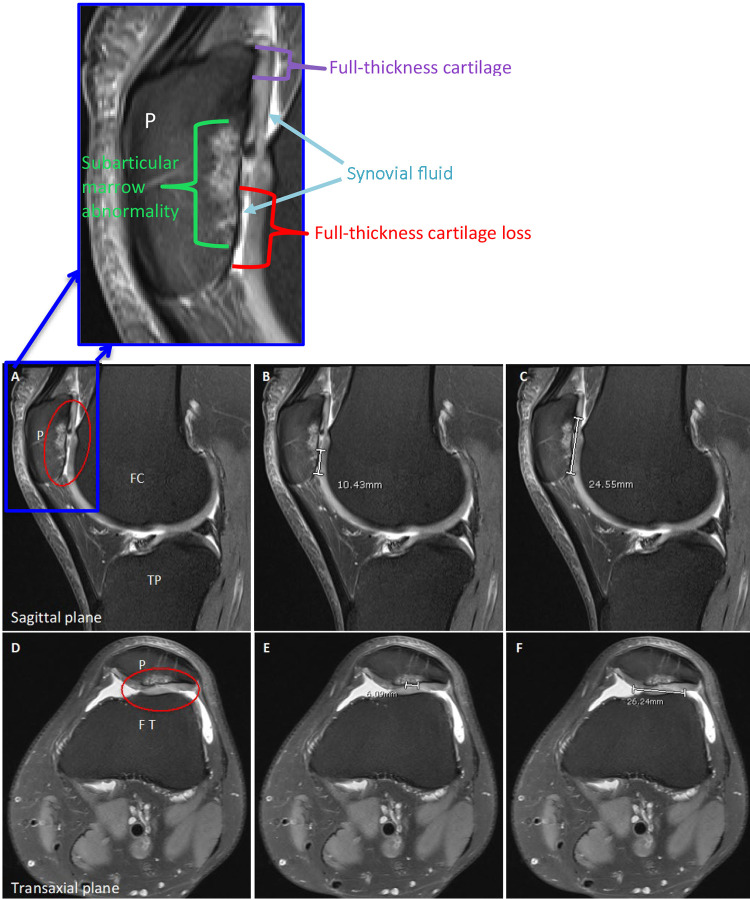
Preoperative 3-T proton density–weighted turbo spin-echo fat-suppressed magnetic resonance imaging scans of a patellar defect in the (A)-(C) sagittal and (D)-(F) transaxial planes. Inset shows the cartilage defect at a higher magnification, indicating abnormalities in the cartilage and bone. Defect areas outlined by a red oval highlight (A) the cartilage degeneration on the lateral patella with (D) matching preserved articular cartilage on the dysplastic femoral trochlea. (B, E) The full-thickness cartilage component of the patellar defect measured 10.4 mm × 6.1 mm (area = 0.6 cm^2^), whereas (C, F) the total predicted abnormal cartilage likely to be removed and treated with ACI was much larger, measuring 24.6 mm × 26.2 mm (area = 6.4 cm^2^; qualifying the patient for ACI^34^). FC, femoral condyle; FT, femoral trochlea; P, patella; TP, tibial plateau.

The MRI scans were independently reviewed by 3 assessors (a senior radiologist with 20 years of experience assessing cartilage defects [B.T.], an orthopaedic surgeon [P.J.], and a medical research scientist with some experience in interpreting MRI scans [J.P.]) to assess both the total area of full-thickness cartilage defect where no cartilage remained and the total abnormal cartilage area that they predicted would be debrided at stage 2. The total predicted abnormal cartilage area was identified as altered signal intensity and/or morphological changes. All cartilage defect area measurements were calculated as the length multiplied by the width of the defect (Figure 1). No training or consensus session for the 3 assessors was provided, aiming to reproduce current clinical practice where no consensus sessions exist and to assess whether there are inherent differences in the assessment by members of different clinical specialties and training.

The defect areas measured by the radiologist were considered to represent the radiological standard for MRI measurements and were used to compare against the areas measured by the surgeon pre- and postdebridement at arthrotomy during stage 2. Postdebridement surgical measurements obtained during arthrotomy were used as the clinical reference standard with which to compare the imaging measurements. Measurements were classified according to defect location: patella, trochlea, medial femoral condyle (MFC), and lateral femoral condyle (LFC).

### Statistical Analysis

Data were tested for normality using the Shapiro-Wilk test, and subsequent analyses were performed as appropriate. The interrater reliability of the MRI-assessed area of full-thickness cartilage defects and total predicted abnormal cartilage area after debridement was determined using the 2-way intraclass correlation coefficient for agreement (ICC(A,1)),^30^ in which ICC values <0.4 were considered poor, between 0.4 and 0.59 fair, between 0.6 and 0.74 good, and above 0.75 excellent.^13,15^ In addition to the overall reliability, we also determined separate reliabilities for each defect location. Mean differences between measurements made on MRI and at arthrotomy were evaluated using a Wilcoxon matched-pairs signed-rank test.

Next, Bland-Altman plots were produced to determine the agreement between defect sizes measured on MRI and during arthrotomy.^6^ Finally, receiver operating characteristic (ROC) curves were determined for the 2 MRI methods, with full-thickness cartilage defect area and total predicted abnormal cartilage area as the predictor and a debrided cartilage area >2 cm^2^ (the NICE criterion for ACI) during arthrotomy as the response. For both MRI methods, we determined the area under the ROC curve (AUC) and the optimal threshold area on MRI to separate patients with defects >2 versus ≤2 cm^2^. The optimal threshold was determined using the Youden criterion on a smoothed ROC curve, and the AUCs were compared using the DeLong test.

Statistical analyses were performed using GraphPad Prism 8 (GraphPad) (normality and matched pair test), R Version 3.6.0 (R Foundation for Statistical Computing) with the packages *irr*, *pROC*, and *cutpointr* (interrater reliability and ROC analysis) and Excel 2011 (Microsoft; Bland-Altman plots). For all statistical analyses, a 2-sided *P* value <.05 was set as the threshold for statistical significance.

## Results

The within-patient differences in area between the methods were distributed normally, whereas the cartilage defect area measurements via the different methods were not.

### Patient Defects

In total, 87 defects were treated in 64 patients (25 left and 39 right knees). The defects were located on the femur (8 LFC, 26 MFC, and 17 trochlea) or patella (n = 36). There were no tibial defects. The patients had received a median of 0 surgical interventions (interquartile range [IQR], 0-1) before ACI.

### Interrater Reliability

**Figure 2.**
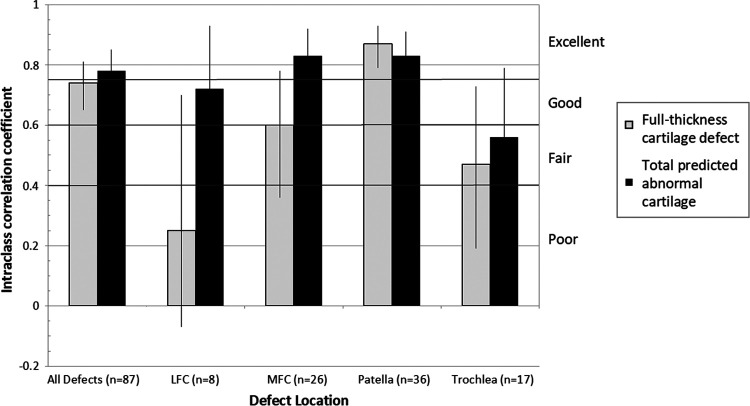
Interrater reliability of measuring the full-thickness cartilage defect and total predicted abnormal cartilage area on preoperative MRI by 3 observers. Black error lines denote 95% confidence intervals. LFC, lateral femoral condyle; MFC, medial femoral condyle; MRI, magnetic resonance imaging.

Interrater reliability of the cartilage defect measurements on preoperative MRI was good to excellent for both the overall full-thickness area (ICC = 0.74; good reliability) and the total predicted abnormal cartilage area (ICC = 0.78; excellent reliability) (Figure 2). When the reliability was analyzed separately for each defect location, patellar defects showed excellent reliability, with an ICC of 0.87 for full-thickness cartilage defects and 0.83 for the total predicted abnormal cartilage area. The MFC showed good reliability for full-thickness cartilage defects (ICC = 0.6) and excellent reliability for the total predicted abnormal cartilage area (ICC = 0.83), while the LFC showed poor reliability overall for full-thickness cartilage defects (ICC = 0.25) and good reliability for the total predicted abnormal cartilage area (ICC = 0.72). Reliability for the trochlea was fair overall for both the full-thickness cartilage defects (ICC = 0.47) and the total predicted abnormal cartilage area (ICC = 0.56).

### Cartilage Defect Measurements

**Figure 3.**
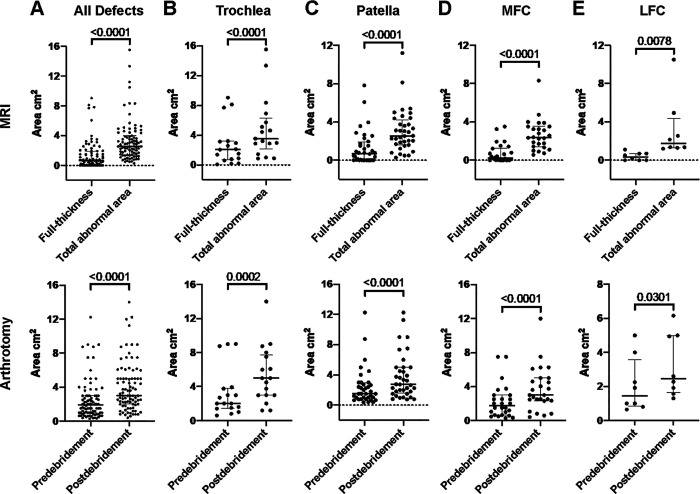
Cartilage defect sizes as measured on MRI (top row) and during arthrotomy (bottom row) by location: (A) all defects, (B) trochlea, (C) patella, (D), MFC, and (E) LFC. The thick horizontal line represents the median, and the error bars represent the interquartile range. On MRI, all defects were significantly larger when the total predicted abnormal cartilage area was measured compared with the loss of full-thickness cartilage, and during arthrotomy, all defects were significantly larger when measured postdebridement compared with predebridement. LFC, lateral femoral condyle; MFC, medial femoral condyle; MRI, magnetic resonance imaging.

On preoperative MRI, the median full-thickness cartilage defect area was 0.65 cm^2^ (IQR, 0.01-1.92 cm^2^), and the median total predicted abnormal cartilage area was 2.57 cm^2^ (IQR, 1.44-4.00 cm^2^) (Table 1). The median total predicted abnormal cartilage area was significantly larger than the median full-thickness cartilage defect area (*P* < .0001); this was the case regardless of the defect location (trochlea [*P* < .0001], patella [*P* < .0001], MFC [*P* < .0001], and LFC [*P* = .0078]) (Figure 3).

**Table 1:** Cartilage Defect Areas Measured on Preoperative MRI Versus during Arthrotomy*^a^*

Defect Location	Defect Area, cm^2^				Difference, %	
	MRI: Full-Thickness Cartilage Defect	MRI: Total Predicted Abnormal Cartilage	Arthrotomy: Predebridement	Arthrotomy: Postdebridement*^b^*	Full-Thickness Cartilage Defect vs Postdebridement	Total Predicted Abnormal Cartilage Versus Postdebridement
All defects (n = 87)	0.65 [0.01 -1.92] (range, 0.00-9.03)	2.57 [1.44-4.00] (range, 0.28-15.51)	1.92 [1.00-3.00] (range, 0.3-12.25)	3.00 [2.00-5.00] (range, 0.4-14.0)	78.3	14.3
Trochlea (n = 17)	2.05 [0.68-3.16] (range, 0.06-9.03)	3.52 [2.14-6.30] (range, 0.84-15.51)	2.00 [1.45-3.84] (range, 0.6-9.0	5.00 [3.00-7.75] (range, 1.2-14.0)	59.0	29.6
Patella (n = 36)	0.71 [0.01 -1.90] (range, 0.00-7.83	2.54 [1.62-4.22] (range, 0.28-11.2)	1.50 [0.85-2.93] (range, 0.36-12.25)	2.75 [1.58-5.00] (range, 0.75-12.25)	74.2	7.64
MFC (n = 26)	0.23 [0.00 -1.26] (range, 0.00-3.51)	2.37 [1.41-3.51] (range, 0.56-8.32)	1.75 [0.64-3.00] (range, 0.3-7.5)	3.00 [2.33-5.00] (range, 0.4-12.0)	92.3	21.0
LFC (n = 8)	0.35 [0.00-0.69] (range, 0.00 -1.1)	1.76 [1.30-4.34] (range, 1.2-10.5)	1.46 [0.85-3.56] (range, 0.64-5.0)	2.44 [1.65-4.99] (range, 1.3-6.16)	85.6	27.9

*^a^* Data are shown as median [interquartile range] unless otherwise indicated. LFC, lateral femoral condyle; MFC, medial femoral condyle; MRI, magnetic resonance imaging.

*^b^* Considered the “true” area.

Measurements obtained at surgery during arthrotomy demonstrated a median defect area of 1.92 cm^2^ (IQR, 1.00-3.00 cm^2^) predebridement and 3.00 cm^2^ (IQR, 2.00-5.00 cm^2^) postdebridement (Table 1). Not surprisingly, the median size of cartilage defects measured during arthrotomy was significantly larger when measured postdebridement compared with predebridement (*P* < .0001). Again, this was the case in all defect locations (trochlea [*P* = .0002], patella [*P* < .0001], MFC [*P* < .0001], and LFC [*P*= .03]) (Figure 3).

When comparing the 2 ways of measuring defect area on MRI, the median full-thickness cartilage defect area underestimated the median postdebridement area measured during arthrotomy by 78.3% across all regions, whereas the median total predicted abnormal cartilage area underestimated the postdebridement area by 14.3%, with some variation between locations (Table 1).

### Agreement Between Measurements on Preoperative MRI Scans and During Arthrotomy

**Figure 4.**
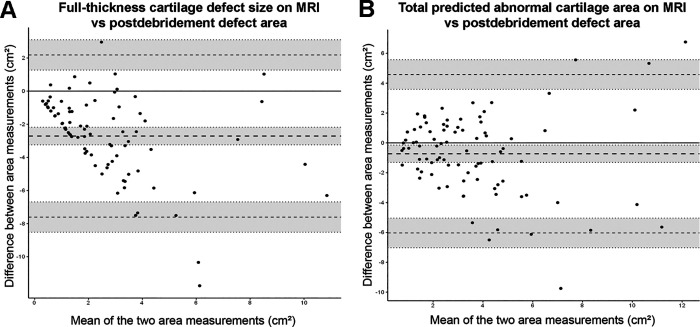
Bland-Altman plots comparing the MRI-measured (A) area of full-thickness cartilage defect size and (B) total predicted abnormal cartilage with the measurement of postdebridement defect size during arthrotomy. In each graph, the horizontal axis represents the mean of the magnetic resonance imaging (MRI) and arthrotomy methods, and the vertical axis the difference between the 2 methods. The horizontal thick dashed line represents the overall mean difference between the 2 methods (bias), the horizontal thin dashed lines represent the 95% limits of agreement (LoA), with the gray error bands representing their 95% confidence intervals. Note that the measurement of the total predicted abnormal cartilage area measured on MRI had a smaller bias (0.7 versus 2.7 cm^2^ when measuring the full-thickness cartilage defect), but that the LoA of the methods were similar.

Bland-Altman plots of the agreement between the cartilage defect sizes measured on MRI and during arthrotomy showed that the variability of the difference with the full-thickness cartilage defect area was consistent over the full measurement range, but the difference with the total predicted abnormal area appeared to increase for larger defect sizes (Figure 4). The bias is represented by the mean difference in area. The full-thickness cartilage defect area measured on MRI underestimated the postdebridement defect area by a mean of 2.7 cm^2^ (Figure 4A), whereas the total predicted abnormal cartilage area measurement underestimated the postdebridement defect area by only 0.7 cm^2^ (Figure 4B), indicating a smaller bias. The limits of agreement for both methods had approximately the same width (±4.9 and ±5.3 cm^2^ for the full-thickness cartilage defect and total predicted abnormal cartilage method, respectively), suggesting a similar level of agreement.

### Sensitivity and Specificity of the MRI Methods in Identifying Defect Areas >2 cm^2^

**Figure 5.**
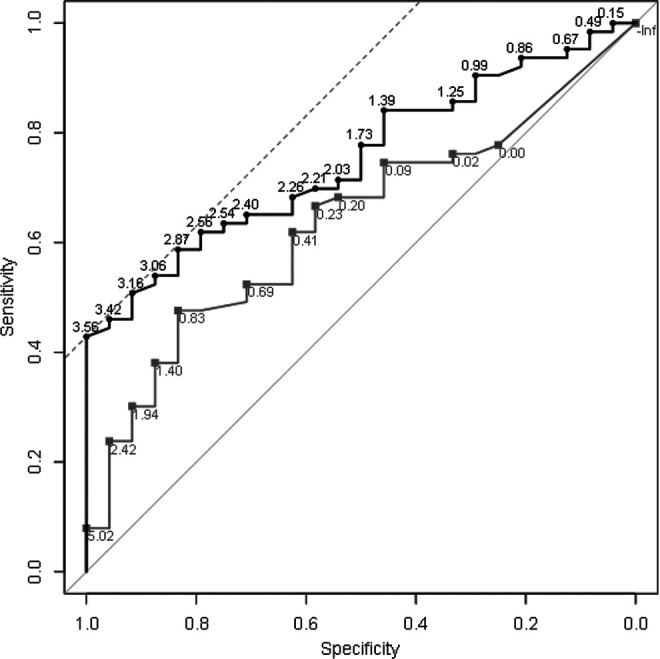
Receiver operating characteristic curve showing sensitivity and specificity at various cut-point areas when using magnetic resonance imaging-determined defect area to identify patients with a defect size >2 cm^2^ (gray curve, full-thickness cartilage defect area; black curve, total predicted abnormal cartilage defect area). The dashed gray line represents the Youden criterion, suggesting several near-equivalent cut-point areas.

Figure 5 shows the results of the ROC analysis to identify which of the 2 MRI methods was more suited to identify patients for ACI. The full-thickness cartilage defect area and total predicted abnormal cartilage method had an AUC of 0.64 (95% CI, 0.51-0.76) and 0.75 (95% CI, 0.65-0.85), respectively, with no significant difference between the methods (*P* = .067). While the full-thickness method had a clear optimal threshold, the abnormal cartilage method had several equivalent ones (Figure 5). We therefore used a smoothed curve to find optimal thresholds, which were 0.98 cm^2^ (44% sensitivity, 83% specificity) for the full-thickness method and 3.33 cm^2^ (46% sensitivity, 92% specificity) for the abnormal cartilage method.

## Discussion

To the best of the authors’ knowledge, this is the first investigational study that compares measuring the full-thickness cartilage defect area and the total predicted abnormal area of cartilage (taking into account perilesional degenerate tissue) on preoperative MRI scans, with the actual size of defect being treated, as measured during arthrotomy (postsurgical debridement). Various methods have been developed to determine the quality and quantity of articular cartilage including both semiquantitative scoring schemes, such as the Whole Organ MRI Score (WORMS) system,^36^ Boston Leeds OA Knee score (BLOKS),^28^ and MRI Osteoarthritis Knee Score (MOAKS),^27^ as well as quantitative methods, such as the Cartilage Lesion Score (CaLS)^1^ that focus on cartilage thickness and volume. While they mostly assess the level of severity of OA in a joint, they do assess the quality of cartilage to some different degrees and the CaLS score provides actual measurements of cartilage defects.^1^ However, none of them address the same issue that is in the current study, that is, the accurate determination of defect size post debridement, which is to be treated with cell therapy such as ACI. This is particularly important in cartilage regeneration surgery, because the choice of technique used is often guided by the size of the defect to be treated as well as influencing the likelihood of reimbursement.^19,26,32,38^

Overall, the interrater reliability was good to excellent for both methods of area measurement on MRI. Since these results were obtained using raters of different backgrounds, they suggest that radiologists, consultant orthopaedic surgeons, and researchers (or other nonclinical staff) can reliably measure the size of cartilage defects with minimal training. Therefore, these measurements can be performed reliably on high-resolution MRI scans by any clinical or nonclinical member of staff, potentially reducing treatment costs and timelines in certain circumstances, for example, clinical trials.

Although the reliability seemed to vary between defect locations, there was minimal evidence that this variation was significant due to the wide confidence intervals around the measurements of the LFC and the trochlea, most likely related to their small sample sizes. The reliability of the full-thickness cartilage defect method and total predicted abnormal area appeared best when measuring patellar defects, perhaps due to the patella having a greater cartilage thickness in comparison with the other anatomical knee joint locations.^17,39^

Another previous study showed that defects with >50% cartilage thinning as assessed on preoperative MRI were actually over 60% larger at the time of cell implantation (postdebridement).^20^ Our study also showed that the median final defect area to be treated with ACI (ie, after debridement to healthy cartilage) was underestimated by 78.3% if measuring the full-thickness cartilage defects on preoperative MRI, whereas it was only underestimated by 14.3% if measuring the total predicted abnormal area. Assuming a preference for using a method without having to adjust for bias, using the total abnormal cartilage area appears superior in terms of predicting defect sizes to be treated when compared with measuring the area of cartilage, with 50% to 100% loss of cartilage thickness. Hence, we recommend surgeons use this method before surgical interventions such as ACI, when the area to be treated may affect reimbursement.

The agreement between measuring the cartilage defect sizes on MRI and during arthrotomy also demonstrated the smaller bias of the abnormal cartilage MRI area measurement, compared with the area of full-thickness defect, which had an approximately 4-fold greater bias (Figure 4). By itself, a bias is not necessarily a problem as one can always adjust for it by subtracting or adding it to the measured value.^6^ In this case, to get corrected numbers, simply add 2.7 cm^2^ to full-thickness cartilage defect sizes and 0.7 cm^2^ to the predicted abnormal cartilage size when measured preoperatively on MRI. However, in clinical practice the obvious problem with adding 2.7 cm^2^ to every defect area measured using the full-thickness method would automatically make every defect larger than the 2 cm^2^ NICE criterion for ACI. In other words, such a simple correction makes the method unsuitable for clinical decisions around smaller defect sizes.

Given that no MRI method is without bias and has variable agreement, measuring defect area during arthroscopy might seem a better approach. However, previous work has demonstrated that arthroscopic measurements of the defect size are also biased and exceed those obtained during arthrotomy by 25% on average, regardless of the defect location (LFC, MFC, patella, and trochlea).^35^ Given that no method is perfect, it seems advantageous to choose MRI over arthroscopy to predict the size of cartilage defects, as it is less invasive with no associated surgical risks. Moreover, if no abnormality or a minor abnormality such as a chondromalacia patella is detected, using MRI can avoid unnecessarily exposing the patient to a surgical treatment with no symptomatic benefit.^16^

While we have shown in this study that using current MRI sequences can predict the median cartilage defect size of a group of patients within 30% of the “true” defect size measured at arthrotomy, this does not apply to individual defects where the differences, as determined by the limits of agreement, are approximately ±5 cm^2^ around the bias for either method. Although advances in new MRI techniques and sequences may improve the assessment in the future, one will currently have to accept that differences between MRI and arthrotomy may be large for individual patients. Results of our ROC analyses (Figure 5) indicated that MRI has a relatively low sensitivity when identifying defects generally considered large enough for ACI treatment (>2 cm^2^)^5^ but a high specificity. Therefore, when faced with borderline defect sizes for multiple repair techniques, we suggest that orthopaedic surgeons discuss the different treatment strategies with the patient and prepare for theatre accordingly, with a strong suggestion in favor of ACI if the defects are over 3.33 cm^2^ based on the abnormal cartilage area, or 0.98 cm^2^ based on the full-thickness defect area.

### Limitations

The main limitations of this study are generally related to the subjective aspects of the defect measurements. However, the interrater reliability for agreement using 3 independent assessors from different disciplines and blinded to each other’s assessments or the true measurements suggested excellent reliability overall. It was not possible to determine the reliability of our clinical reference standard (the measurements made during arthrotomy) which would involve another surgeon replicating them. We are also not aware of any published work looking at the reliability of such measurements, although they have also been used as the clinical gold standard in other studies.^2,35^ While only defects on the MFC, LFC, trochlea, and patella were considered in this study, we would expect the results to be broadly similar with other areas of joints to be treated, for example, on the tibia.

Moreover, it should be noted that 2 surgeons performed the surgeries and also used 2 different tools (caliper and/or a ruler) to measure the cartilage defects during arthrotomy, introducing the risk of further inconsistencies between these measurements. Another limitation is the fact that defect sizes may progress between preoperative MRI and the cell implantation stage. In this patient cohort, the maximum gap was 9.1 weeks, which is probably not long enough for the defect to increase significantly, in light of research suggesting that cartilage volume loss occurs at a rate of <5% annually in both men and women.^8,22^ Finally, all defect areas were measured as the length × width and expressed as an area in square centimeters, as performed in routine clinical practices and widely throughout the literature.^4,20^ However, we are aware that this method does not take into account the exact shape of the cartilage defects measured.

## Conclusion

Measurements of both full-thickness cartilage defect size and total predicted abnormal cartilage on preoperative MRI can be reliably obtained by any adequately trained personnel, who need not necessarily be medically qualified. From our findings, we suggest that for planning surgical treatment of cartilage defects, the total predicted abnormal cartilage area, rather than just the full-thickness cartilage defect component, should be assessed on preoperative MRI in order to be closer to the “true” chondral/osteochondral defect size that will ultimately be treated.
